# Platelet-Derived Exosomes in Atherosclerosis

**DOI:** 10.3390/ijms232012546

**Published:** 2022-10-19

**Authors:** Chiara Gardin, Letizia Ferroni, Sara Leo, Elena Tremoli, Barbara Zavan

**Affiliations:** 1 Maria Cecilia Hospital, GVM Care & Research, 48033 Cotignola, Ravenna, Italy; 2 Department of Translational Medicine, University of Ferrara, 44121 Ferrara, Ferrara, Italy

**Keywords:** atherosclerosis, cardiovascular diseases, platelets, platelet-derived exosomes, biomarkers, miRNAs

## Abstract

Atherosclerosis (AS), the main cause of many cardiovascular diseases (CVDs), is a progressive inflammatory disease characterized by the accumulation of lipids, fibrous elements, and calcification in the innermost layers of arteries. The result is the thickening and clogging of these vessel walls. Several cell types are directly involved in the pathological progression of AS. Among them, platelets represent the link between AS, inflammation, and thrombosis. Indeed, besides their pivotal role in hemostasis and thrombosis, platelets are key mediators of inflammation at injury sites, where they act by regulating the function of other blood and vascular cell types, including endothelial cells (ECs), leukocytes, and vascular smooth muscle cells (VSMCs). In recent years, increasing evidence has pointed to a central role of platelet-derived extracellular vesicles (P-EVs) in the modulation of AS pathogenesis. However, while the role of platelet-derived microparticles (P-MPs) has been significantly investigated in recent years, the same cannot be said for platelet-derived exosomes (P-EXOs). For this reason, this reviews aims at summarizing the isolation methods and biological characteristics of P-EXOs, and at discussing their involvement in intercellular communication in the pathogenesis of AS. Evidence showing how P-EXOs and their cargo can be used as biomarkers for AS is also presented in this review.

## 1. Introduction

Atherosclerosis (AS) is a chronic inflammatory disease characterized by the deposition of lipids, fibrous material, and calcium in the inner layers of the large and medium arteries [1]. As this material builds up, the artery walls become stiff and thick, reducing the internal diameter of the vessel and therefore reducing blood flow. AS underlies most cardiovascular diseases (CVDs), such as myocardial infarction (MI), ischemic stroke, and peripheral vascular disease, the main causes of death and disability worldwide [2].

The molecular events in AS include endothelial dysfunction, fatty streak formation, cell migration into the vessel wall, foam cell formation, atherosclerotic plaque development, and rupture [3,4]. Endothelial cells (ECs), leukocytes (monocytes, neutrophils, T lymphocytes, and dendritic cells), platelets, and vascular smooth muscle cells (VSMCs) participate in the above cascade of events by actively communicating with each other [5].

Platelets are key players in the atherosclerotic process, and are now believed to provide the link between AS, inflammation, and thrombosis [6]. In fact, they participate in the formation of blood clots following the rupture of the plaque, and modulate the immune response at the sites of the lesion [7]. Activated platelets receive inflammatory signals from neighboring cells, and simultaneously release a multitude of cytokines and chemokines that promote the activation and recruitment of leukocytes, ECs and VSMCs, thus boosting the inflammatory milieu [8].

It the last couple of decades, the discovery of extracellular vesicles (EVs) has represented one of the most revolutionary contributions to cell biology, and the transfer of EVs has been recognized as a central mechanism of intercellular communication [9]. EVs are bilayer membrane vesicles that can differ in size, density, content, morphology, and protein composition. They are named by size as apoptotic cell-derived EVs (ApoEVs, >1000 nm), microparticles (MPs, 100–1000 nm), also called microvesicles, and exosomes (EXOs, 30–100 nm) [10]. ApoEVs are released via plasma membrane blebbing during apoptosis, and can be engulfed by target cells to promote tissue regeneration, embryonic development, immune regulation, and cancer development [11]. Instead, MPs are derived from activated or apoptotic cells through the outward budding and fission of the plasma membrane at lipid-rich microdomains. The outward budding can depend on different enzymes and mitochondrial or calcium signaling in response to several stimuli such as hypoxia, irradiation, oxidative injury, apoptosis, or shearing stress. Almost all mammalian cells can release MPs including blood cells (e.g., platelets, erythrocytes, leukocytes), VSMCs, and ECs [12]. Lastly, EXOs are the EVs of endosomal origin that can be secreted by any cell type both in physiological and pathological conditions [13]. EXOs have been identified in many biological fluids including saliva, plasma, serum, urine, amniotic fluid, breast milk, and seminal fluid. EXOs are formed by the inward budding of the membrane of early endosomes, which then mature into multivesicular bodies (MVBs). MVBs can eventually fuse with the plasma membrane to release its content, including EXOs, into the extracellular space or be degraded into lysosomes. The complex machinery of proteins and lipids that regulate the biogenesis and release of EXOs has already been extensively described elsewhere [14]. Briefly, EXO generation is regulated by endosomal sorting complex required for transport (ESCRT), the tetraspanin proteins (e.g., CD63, CD9, CD81), and ceramide. As ESCRT proteins, their accessory proteins (Alix, TSG101, HSC70, and HSP90β), and the aforementioned tetraspanins are involved in EXO formation, these proteins are commonly considered exosomal marker proteins.

Despite a different biogenesis, both MPs and EXOs carry bioactive molecules (DNA, mRNAs, noncoding RNAs, proteins, and lipids), and have the ability to transfer them to target cells, thus acting as cell-to-cell messengers in the human body [5]. Most of the circulating MPs derive from platelets (P-MPs), where they were first identified by Wolf and colleagues, who called them “platelet dust” having pro-coagulant activity [15]. Later on, it was found that EVs generated by platelets also include EXOs (P-EXOs) [16].

Increasing evidence suggests the involvement of EVs in AS, as high concentrations of vesicles have been measured in patients with CVDs and within the atherosclerotic plaque [17,18]. Actually, EVs are thought to participate throughout the atherogenic process because vesicles have been found in both developing and advanced plaques [19].

While the number of publications on the involvement of P-MPs in AS and atherothrombosis has dramatically increased in the past few years [20,21,22,23,24,25,26], relatively little is known about the roles of P-EXOs in the same processes. In this review, we first briefly summarize the atherosclerotic process with an emphasis on platelets; then, we provide updated information on P-EXOs, including the currently used methods for their isolation and characterization. Lastly, we present the latest evidence on P-EXO involvement in AS, focusing on their role as predictive biomarkers.

## 2. Brief Overview of the Pathogenesis of AS

The pathogenic mechanism of AS is extremely complex and articulated. For a complete overview of AS pathophysiology, we recommend a couple of valuable papers [1,20]. Nonetheless, a brief description of the process is imperative for a better comprehension of this review.

Conventionally, AS can be divided into three consecutive phases: initiation, progression, and complications [27]. The process of AS is thought to begin when the low-density lipoprotein (LDL) cholesterol circulating in the blood is accumulated and oxidized in the intima, the innermost layer of the artery wall, leading to EC activation [28]. Activated ECs express high levels of E- and P-selectins, adhesion molecules (e.g., intercellular adhesion molecule-1 (ICAM-1), vascular cell adhesion molecular-1 (VCAM-1)) and release a large amount of chemokines and cytokines that attract leukocytes. The contact of activated ECs with platelets contributes to the rolling of leukocytes along the endothelium through a loose bond, mediated by selectins. This activates the leukocyte integrins that bind ICAM-1 and VCAM-1 of the activated ECs. This results in the arrest of leukocytes on the endothelium and subsequently in the passage of them through the endothelial barrier [29]. Therefore, the activation of ECs represents the initiation of a robust inflammatory reaction that results in the recruitment of neutrophils, monocytes, and, to a lesser extent, T lymphocytes to the intima [30]. Circulating neutrophils adhere to the EC surface, interact with platelets, and assist in the migration of monocytes and T lymphocytes. Once monocytes are recruited to the intima, they differentiate into macrophages and internalize the oxidized-LDL (ox-LDL) particles through scavenger receptors (SRs), becoming foam cells. The foam cells secrete local cytokines, such as platelet-derived growth factor (PDGF), interleukin-1 β (IL-1β), interferon-γ (INF-γ), tumor necrosis factor-α (TNF-α), and transforming growth factor-β (TGF-β), that promote the proliferation and migration of VSMCs from the middle layer into the intima. T lymphocytes also localize within the lesions where they can assume different programs of activation, switching from pro-inflammatory (Th 1) to anti-inflammatory (Th 2, Treg) cells, thereby positively or negatively influencing atherosclerotic plaque progression [27,31].

The progression of atherosclerotic lesions coincides with the production by the recruited VSMCs of extracellular matrix (ECM) molecules, including collagen, elastin, fibronectin, proteoglycans, and glycosaminoglycans, that contribute to the formation of a fibrous cap. Many of these ECM molecules have the ability to entrap lipoproteins, thus enhancing lipid accumulation and the thickening of the intimal layer. While atherosclerotic plaque advances, macrophages and VSMCs can undergo apoptosis, forming the lipid-rich necrotic core of the plaque. The impaired removal of dead cells, known as defective efferocytosis, contributes to the growth of this necrotic core [32]. During its evolution, an atherosclerotic plaque may develop regions of calcification deriving from the dysregulation of calcium deposition and impaired clearance [33]. Although calcification is a hallmark of advanced AS, the amount and size of calcium deposits do not reflect plaque stability, which is rather associated with other characteristics, such as calcification type, location, or the surrounding environment [34].

Complications of AS arise when the macrophages and T lymphocytes confined at the edges of the plaque secrete reactive oxygen species (ROS) and matrix metalloproteinases (MMPs) that degrade the ECM and inhibit VSMC proliferation and collagen synthesis. This weakens the fibrous cap, making it susceptible to rupture [35]. The plaque rupture exposes the constituents of the basal lamina, and this attracts circulating platelets. When the macrophages release the powerful pro-coagulating tissue factor (TF) in the site of the rupture, the clotting process begins, leading to the formation of an occlusive thrombus. Thrombosis is eventually the cause of clinical manifestations and ischemic cardiovascular disease, such as MI and stroke [36].

It is important to highlight that not all of the atherosclerotic plaques are unstable and evolve toward rupture. Some plaques possess a stable fibrous cap that provides an effective barrier, preventing plaque rupture and the exposure of the lesion matrix and pro-thrombotic factors, thereby weakening the likelihood of thrombus formation and the manifestation of clinical events [37].

Besides hypercholesterolemia, other important risk factors implicated in AS include hypertension, obesity, diabetes, smoking, an inactive lifestyle, and age [38]. Hypertension increases shear forces at arterial branches or points of curvature, whereas diabetes leads to the formation of advanced glycation end products. Smoking introduces chemical irritants into the arteries that can directly induce endothelial dysfunction and enhance the inflammatory response [31,39].

## 3. Role of Platelets in AS

Platelets, or thrombocytes, are anucleated cell fragments of approximately 2 µm in diameter deriving from large bone-marrow-derived cells called megakaryocytes [39]. The involvement of platelets in AS was initially described in the context of thrombosis, the event following the rupture of an atherosclerotic plaque. Later, it became clear that platelets also have the ability to influence the early stages of the atherosclerotic process as well as the stability of atherosclerotic plaques by modulating the function of other blood and vascular cell types [7].

During the early stage of AS, platelets participate in binding the dysfunctional endothelium, thus acting as a link between ECs and leukocytes. The first contact between circulating platelets and the vascular endothelium, a phenomenon known as platelet rolling, is mediated by P-selectins, which are surface glycoproteins of both platelets and ECs. In particular, P-selectin on ECs binds to the glycoprotein Ibα (GPIbα) on the membrane of platelets, whereas the platelet P-selectin interacts with the endothelial P-selectin glycoprotein ligand-1 (PSGL-1). Since these bindings are insufficient for stable adhesion, additional contacts between platelets and the endothelium occur [40]. Integrins αIIbβ3, α5β1, α2β1 on platelets, as well as fibronectin, fibrinogen/fibrin, and collagen on ECs allow firm platelet–endothelium adhesion [41]. Such combined interactions result in the activation of platelets that release into the local environment the content of their granules, consisting of a vast array of cytokines, chemokines, growth factors, and coagulation factors. All these molecules contribute to the modulate adhesion, chemotaxis, proliferation, and proteolysis of ECs, activities that in turn accelerate the recruitment of inflammatory cells at lesion sites. Just to report some examples, platelet-derived IL-1β induces the secretion of cytokines IL-6 and IL-8, and the expression of ICAM-1 and monocyte chemoattractant protein-1 (MCP-1) by ECs. Additionally, the binding of platelet CD40 ligand (CD40L) to CD40 on ECs results in the up-regulation of ICAM-1, VCAM-1, E- and P-selectin, IL-6, IL-8, and TF release, as well as the inhibition of nitric oxide (NO) synthesis [41,42]. Through these mechanisms, platelets significantly alter the chemotactic and adhesive properties of ECs that enhance monocyte and neutrophil adhesion to the endothelium.

Activated platelets have also been found to aggregate with circulating leukocytes, generating leukocyte–platelet aggregates (PLAs) [43]. Platelets communicate with monocytes, neutrophils, dendritic cells, and T lymphocytes through adhesive receptors and secreted mediators [44,45,46]. Just like platelet–EC interactions, the recruitment of leukocytes is also a well-controlled multistep process, starting with the binding of the platelet P-selectin to its receptor analogue on leukocytes, PSGL-1. This interaction culminates in the activation of the leukocyte β2 integrins macrophage antigen-1 (Mac-1) and lymphocyte function-associated antigen-1 (LFA-1), which are necessary for stable leukocyte adhesion [42]. As a result, platelets secrete chemokines, such as CCL-5 (or RANTES) and CXCL-4 (or platelet factor 4, PF4), which in turn lead to enhanced monocyte adhesion at injury sites. Monocytes then migrate towards the endothelium and differentiate into macrophages, which internalize lipids and become foam cells, as described earlier.

It is important to highlight that the crosstalk between platelets and ECs or between platelets and leukocytes is often bidirectional. Indeed, factors released by leukocytes, including proteases and NO, can further modulate platelet responses [43].

In addition, some studies have investigated the interactions between platelets and components of the vascular wall. Precisely, PF4 (CXCL-4) secreted by platelets drives the recruitment of VSMCs towards injury sites, promoting their proliferation and switch into an inflammatory phenotype characterized by increased cytokine production [47,48].

Another way through which platelets play an active role in AS is by binding and transporting native and modified lipoproteins [49]. The binding of native LDL to platelets leads to the activation of signal transduction pathways that induce the resynthesis or remodeling of phospholipids in the cell membrane. Native LDL also alters platelet activity by the insertion of phospholipids from circulating lipoproteins to platelets and other cells, thus modifying the composition of membrane phospholipids, whereas the ox-LDL binds to resting platelets through CD36, a glycoprotein receptor constitutively expressed on platelets [50]. This interaction activates platelets, inducing the expression of P-selectin and CD40L, and the activation of integrin αIIbβ3, which is a receptor for fibrinogen, thus promoting the formation of platelet–leukocyte complexes [51]. Another important binding protein for ox-LDL on platelets is lectin-like oxidized low-density lipoprotein receptor-1 (LOX-1) [52]. Binding of ox-LDL to LOX-1 leads to the activation of integrins αIIbβ3 and α2β1, which are receptors for fibrinogen and collagen, respectively. These interactions encourage a fast change in platelet shape and aggregation that contributes to thrombus formation following the plaque rupture. The binding of ox-LDL to activated platelets through LOX-1 also results in ROS production that, in turn, determines the further oxidation of LDL and, consequently, platelet activation [53].

Besides pro-thrombotic effects, platelet ligation to ox-LDL also exerts pro-atherogenic functions, as demonstrated by the enhanced release of chemokines and cytokines from ECs that in turn activates inflammatory cells, and by the increased expression of adhesive proteins. In addition, ox-LDL-laden platelets directly participate in foam cell formation by mediating phagocytosis by macrophages [54,55].

**Figure 1:**
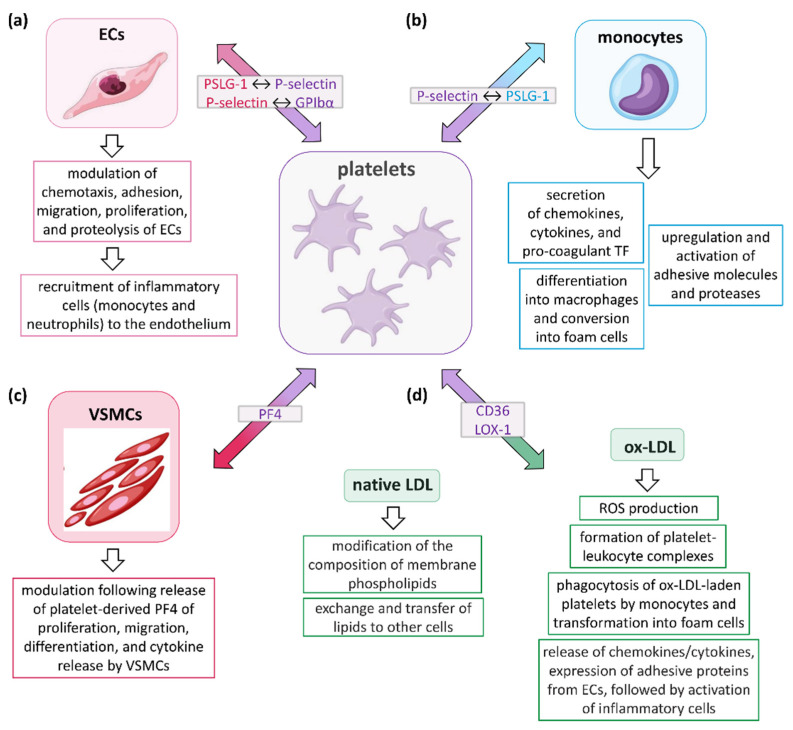
Role of platelets in the development and progression of AS. Platelets influence the atherosclerotic process by communicating with (**a**) endothelial cells (ECs), (**b**) leukocytes (mainly monocytes, but also neutrophils, T lymphocytes, and dendritic cells), (**c**) vascular smooth muscle cells (VSMCs), and (**d**) low-density lipoprotein (LDL), either in the native or oxidized form (ox-LDL). Collectively, these interactions induce platelets to release a variety of inflammatory mediators capable of affecting several biological functions of their target cells, including secretion, adhesion, migration, recruitment of other cell types, proteolysis, and coagulation. In the light grey boxes, the molecules participating in initial interactions between cells are indicated, with the colors representing the cells in which they are expressed. PSLG-1, P-selectin glycoprotein ligand-1; GPIbα, glycoprotein Ibα; TF, tissue factor; PF4, platelet factor 4 (or CXCL-4); LOX-1, low-density lipoprotein receptor-1; ROS, reactive oxygen species.

The roles of platelets in relation to the different cell types participating in the development and progression of AS are schematically illustrated in Figure 1.

## 4. Platelet-Derived Exosomes

Platelet derivatives, such as platelet-rich plasma (PRP) or platelet lysate (PL), have been widely used in the field of regenerative medicine over the past 30 years [56]. Therapeutic applications of platelet concentrates have been described in dermatology, plastic surgery, oral, maxillofacial and orthopedic surgery, pain management, musculoskeletal and neural regeneration, and CVDs, among others [57,58,59,60,61]. The regenerative potential of such platelet derivatives is generally attributed to the supra-physiological concentration of growth factors, cytokines, chemokines, and adhesion proteins [62]. All these molecules are released upon platelet activation and directly contribute to tissue repair and regeneration [63].

Although PRP- and PL-based therapies have shown promising results, some concerns and limitations regarding their clinical use exist. First, there is no standardization for the classification of platelet derivatives, nor for their preparation, as more than 20 different commercial separation systems are currently available [64]. Further, the heterogeneity between biological formulations is related to the high variability among donors and the storage conditions prior to use. Additionally, there is no consensus on the optimal platelet concentration or the timing of injections for the various therapeutic indications [65]. All of these aspects are reflected in the lack of reproducibility among platelet-based therapies and, thus, in clinical outcomes.

In light of the above considerations, platelet-derived EVs (P-EVs) could represent a promising alternative to such platelet concentrates, and even present some desirable advantages that could improve the benefits of their clinical use. P-EVs, comprising both P-MVs and P-EXOs, are actually considered the true effectors of platelet derivatives and platelets themselves [66].

One of the main advantages of P-EXOs relies on their ability to carry a multitude of bioactive molecules and to protect this cargo from degradative enzymes or chemical agents. Consequently, the molecules transported by P-EXOs retain their biological activity longer once exposed to the extracellular environment [67]. In addition, the small size of P-EXOs likely contributes to their stability in circulation and is beneficial for their transfer across biological barriers, such as the blood–brain barrier. A direct implication is that P-EXOs may represent potential carriers for the delivery of drugs or molecules of therapeutic interest, especially to bodily regions where platelets are rarely found [68]. Noteworthily, P-EXOs can be easily administered through a variety of non-invasive routes to increase bioavailability depending on the clinical purpose. Additionally, thanks to their resistant membrane, P-EXOs maintain their integrity after freeze–thaw cycles, making long-term storage without biological degradation possible, and overcoming the current limitations of transporting and using fresh platelet derivatives [67].

When compared with EXOs from other sources, especially stem cells, P-EXOs possess some additional advantages. First, isolation of P-EXOs does not require upstream cell expansion, which is instead indispensable for EXOs from other cells, since these vesicles can be directly extracted from platelet derivatives [69]. In addition, platelets allow for higher amounts of EXOs to be obtained using minimally invasive procedures, as blood is easily accessible, routinely isolated, and it represents the most relevant body fluid for cardiovascular applications [70]. Importantly, given the anucleated nature of platelets, P-EXOs are not tumorigenic. These characteristics, together with the non-cytotoxicity and the non-immunogenicity, alleviate the safety concerns regarding possible risks associated with the clinical use of P-EXOs [71].

While EXOs from nucleated cells have been extensively studied in recent years, only a few works so far have considered the existence and biological activity of EXOs derived from platelets. In the following paragraphs, we describe the currently reported methods for the isolation and characterization of P-EXOs.

### 4.1. Methods for Isolation of P-EXOs

Théry and colleagues reported the first work on the isolation of EXOs from platelets in 2006 [72]. Actually, the authors described a method for purifying EXOs from serum and plasma, referring to a study published a year earlier by the same research group and based on differential centrifugation (DC) [73]. The DC methodology allows the isolation of vesicles according to their size and density by consecutively increasing the centrifugal force [74]. Plasma is usually preferred over serum for EXO isolation because serum releases additional vesicles during in vitro clot formation, thus resulting in a sample that does not completely represent the original vesicle composition [70]. To obtain plasma, blood needs anticoagulant, the choice of which is strongly dependent on the downstream analysis. For example, ethylenediaminetetraacetic acid (EDTA) is suitable for RNA analysis, unlike heparin that inhibits the activity of polymerase during the PCR reaction, whereas citrate–dextrose solution (ACD) is preferred for in vitro study [75,76]. The International Society on Thrombosis and Hemostasis (ISTH) recommends sodium citrate as an anticoagulant, thanks to its ability to reduce in vitro platelet activation and subsequent P-EV release [77]. Indeed, since platelets are easily activated and secrete vesicles during sample manipulation, the P-EXO isolation protocol should prevent platelet activation. Other general recommendations for limiting platelet activation include the use of a large needle for blood collection, and the elimination of the initial 2–3 mL of the collected blood [78]. Moreover, as a general rule, the time interval between blood collection and the first step of plasma preparation should be minimized in order not to adversely affect the concentration and functionality of the isolated P-EXOs [70]. To date, there is no ideal storage condition for the isolated P-EXOs and their shelf life. Most of the published articles suggest to snap-freeze aliquots in liquid nitrogen, store them at −80 °C, and then thaw at 37 °C before use [70,79].

With these indications in mind, the reported methods used for the isolation of P-EXOs can be grouped into three categories: (i) direct purification from activated PRP [71,76,80,81]; (ii) isolation from the supernatants of activated platelets previously separated from PRP [82,83]; (iii) isolation from the lysates of non-activated platelets [84].

Regardless of the starting sample, P-EXOs are principally isolated from platelet derivatives by DC. It should be stressed that, as it is not currently possible to establish a universal protocol between different laboratories using the DC method, adequate reporting of the parameters used for P-EXO isolation is essential. In particular, at least the type of rotor and its associated k-factor must be declared in the published article [85,86].

Direct purification from activated PRP is currently the most widely used technique to isolate P-EXOs. This method, which assumes that most of the EXOs present in PRP derive from platelets, can be summarized into three key steps: (i) PRP is prepared from whole blood; (ii) PRP is activated to promote platelet vesiculation; (iii) P-EXOs are isolated by DC. PRP is generally obtained by low-speed centrifugation of whole blood in the presence of an anticoagulant followed by higher speed centrifugation to allow the sedimentation of platelets [76,80]. Alternatively, PRP can be obtained by using a fully automatic blood separator, equipped with a leukocyte-reduction system to avoid leukocyte contamination [81]. Platelet concentrates are then activated with specific agonists in order to stimulate the release of P-EXOs. The most popular activators include thrombin, collagen, calcium ionophore, calcium chloride, calcium gluconate, ADP, and thrombin receptor-activating peptide-6. It has been demonstrated that diverse platelet activators differently affect the quality and quantity of the P-EXO subpopulation, with thrombin and calcium gluconate together yielding the highest P-EXO concentration and cytokines with respect to thrombin or calcium gluconate alone [81]. EXOs can be isolated and purified from activated PRP by the method based on DC described by Théry and colleagues, with minor modifications [72]. Otherwise, P-EXOs can be purified using commercial kits such as the exoEasy Maxy Kit (Qiagen, Hilden, Germany) [80], and the ExoQuick™ Exosome Precipitation Solution (System Biosciences, Palo Alto, CA, USA) [87].

The isolation of P-EXOs from supernatants of activated platelets also follows Théry’s procedure, with some modifications [72]. The resuspended activated platelet pellet is subjected to a series of low-speed centrifugation steps, ultrafiltration, and then ultracentrifugation onto a 30% sucrose–D_2_O cushion to purify the P-EXOs [82,83].

Lastly, P-EXOs can be purified from the lysates of non-activated platelets, the so-called PL. The PL is a cell-free supernatant generated by a simple freeze–thaw procedure of platelet units derived from apheresis [88]. To our knowledge, only one published study reports EXO isolation from human PL by serial low-speed centrifugations followed by ultracentrifugation [84].

In general, the isolation methods based on centrifugation and ultracentrifugation require operator skills, extended execution time, and expensive instrumentation, which not all laboratories can afford. In contrast, commercial kits have been designed to overcome these concerns. However, it should be taken into account that the pool of isolated P-EXOs is closely linked to the isolation method followed. As there are no universally accepted guidelines for the isolation of P-EXOs to date, operators select the most appropriate method based on the experimental conditions, objectives, and laboratory tools.

### 4.2. Characterization of P-EXOs

The characterization of P-EXOs involves the evaluation of multiple parameters, both qualitative (i.e., morphology, size, immunophenotype, nucleic acid and protein contents) and quantitative (i.e., concentration) [89]. Currently, there is no single technique capable of analyzing all of these properties; rather, a complete characterization of the EXO population requires the combination of different techniques. The standard regulations of the International Society for Extracellular Vesicles (ISEV) recommend that at least two different technologies must be used to identify EXOs in order to minimize the likelihood of characterizing co-isolated non-vesicular components [10].

Transmission electron microscopy (TEM) is the gold standard technique for imaging EXOs, and gives information on their morphology, size, and phenotype [90]. TEM allows the monitoring of the quality and purity of samples containing EXOs by discriminating them from similar-sized non-EXO particles, thereby revealing the presence of EXO aggregates [70]. However, due to dehydration and fixation during sample processing, the vast majority of the negatively stained EXOs typically exhibit a cup-shaped morphology. To avoid such artifacts, cryo-electron microscopy (cryo-EM) can be used as an alternative to conventional TEM. Cryo-EM enables the analysis of EXOs in fresh-frozen samples without the need for staining or chemical fixation [91]. Under cryo-EM, EXOs generally show a spherical appearance. When coupled to labeling with colloidal gold particles that are linked to antibodies directed to specific EXO surface antigens, cryo-EM allows the immunophenotypization of EXOs [92].

The morphology of EXOs derived from platelets were observed by TEM in most of the published studies, all confirming the typical cup-shaped appearance of the isolated particles [76,80,81,82,83,84,93]. TEM has also been used to evaluate the size of these particles, which falls in the 30–100 nm range. Interestingly, both the shape and size of EXOs appear to be influenced by the agonist used to activate PRP. For example, EXOs isolated from PRP exposed to calcium gluconate have rougher surfaces and are significantly larger than when using other activators, such as thrombin [81].

The presence and the amount of one or more proteins on the surface of EXOs, or inside them, can be evaluated through several methodologies. The most popular is flow cytometry (FC), which allows the analysis of thousands of vesicles in one sample and simultaneously determines multiple markers, thus quantifying and classifying the vesicle population according to the level of antigen expression [94]. However, the main limitations of FC are the low sensitivity and low resolution for particles smaller than 500 nm in diameter, which means that a significant amount of EXOs is not detected. On the other hand, high concentrations of EXOs may result in the identification of multiple vesicles as a single event, the so-called swarm artifact [95]. Another issue related to EXO analysis by FC concerns the determination of their size [96]. Several modifications of conventional FC have been introduced over the years. One of these is based on the use of micrometer-sized magnetic or latex beads coated with antibodies against membrane antigens that are able to bind to multiple vesicles. Caby and colleagues, for example, used this approach to reveal the expression of the tetraspanins CD9, CD63, and CD81 on EXOs isolated from human plasma. Through the same technique, the presence of the platelet-specific marker CD41 (GPIIb) on P-EXOs was also detected [73]. Although FC of bead-bound EXOs allows for the analysis of EXOs using antibodies that precisely detect these vesicles from heterogeneous samples, this method cannot distinguish between different subpopulations of EXOs, thus resulting in the loss of distinctive signatures [89].

Therefore, other high-resolution FC-based methods are now being developed, which aim to improve the single-vesicle-profiling capabilities. A relatively new technology is imaging FC, which combines the properties of standard FC with high-resolution imaging at the single-cell level. Through this approach, EXOs can be clearly distinguished from beads, cell debris, and parental cells. Additionally, the technique has a high sensitivity for the fluorescence detection of smaller particles, thus enabling the analysis of vesicles with a diameter less than 300 nm [96]. Nanoscale fluorescence analysis and cytometric sorting (NanoFACS) recently emerged as another high-resolution FC-based methodology [97]. This approach combines measurements from high-sensitivity multi-parametric scattered light and fluorescence to analyze and sort EXOs individually. Compared to standard FC, nanoFACS can separate and distinguish the nano-sized particles from instrument noise and background. The technique is also able to discriminate actual EXOs from other nanoparticles, contaminants, or artifacts, providing information on their size, concentration, and distribution in the analyzed sample.

To our knowledge, the above-described FC-based methodologies have not yet been applied to the study of EXOs derived from platelets. More often, classical Western blotting (WB) is adopted to demonstrate the presence of target proteins that are reportedly associated with EXOs. Although WB is time-consuming, it can prove the presence of tetraspanins (CD9, CD63, and CD81), heat shock protein 101 (HSP101), and tumor susceptibility gene 101 (TSG101) [80,83,84,93]. In addition, P-EXOs have been found to be positive for the platelet-specific marker CD41, and negative for the endoplasmic reticulum membrane protein calnexin [81,82,98].

The quantification of EXOs still represents a major challenge. Studies first estimated EXO amounts by measuring the total protein content [99]. However, contamination with high-molecular-weight proteins that co-purify with the isolated EXOs leads to the overestimation of their number; additionally, protein content per vesicle may differ between EXO subtypes. To address this challenge, several other methodologies have been developed for determining EXO concentration. Measurements of particle concentration are now often executed by light-scattering technologies, such as dynamic light scattering (DLS), nanoparticle-tracking analysis (NTA), high-resolution FC, resistive pulse sensing (RPS) or, more recently, tunable resistive pulse sensing (TRPS), or by other techniques with similar features such as atomic force microscopy (AFM) [10].

The DLS technique has been used to assess the size and distribution of P-EXOs in some studies [82,83,93]. DLS measures the scattered light from EXOs when they are passed through a monochromatic laser beam. The DLS analysis is relatively easy, fast, and requires low sample volumes, but reliable data can only be obtained for monodispersed vesicle samples. In the case of polydispersed suspensions, larger particles scatter more light, thus obscuring the signal from the smaller particles. To overcome this problem, the depletion of any large contaminants before analyzing small nanoparticles is highly suggested.

NTA allows such measurements by combining the properties of laser light-scattering microscopy and Brownian motion [100]. NanoSight instruments (Malvern, UK) are currently the most widely used instruments for NTA studies in the EV field, which enable the characterization of particles ranging from 1 to 2000 nm [101]. Unlike DLS, NTA also allows accurate results to be obtained in the case of polydispersed samples. On the other hand, NTA instruments and measurements are less user-friendly and require several optimization steps by a skilled operator [102].

The characterization of protein cargo in P-EXOs has mostly been executed through WB or, in a lower number of studies, by means of sandwich ELISA immunoassays. In both cases, the growth factors more represented in P-EXOs are PDGF-BB, TGF-β, basic fibroblast growth factor (bFGF), and vascular endothelial growth factor (VEGF) [81,82,83,84]. EXOs contain different forms of RNA, mostly represented by miRNAs. MiRNA profiling has revealed that the most highly expressed miRNAs in P-EXOs are miR-21, miR-22, miR-25-3p, miR126-3p, miR-185, miR-223, miR-320b, miR-328, and miR-339 [25,103]. The use of multiple prediction algorithms, such as TargetScan and miRDB, predicted 1453 target genes of the identified miRNA signature. Further bioinformatic analysis highlighted that P-EXOs are enriched for miRNAs that regulate key signaling networks, including the WNT, TGF-β, Hippo, nuclear factor kappa B (NF-kB), and mitogen-activated protein kinase (MAPK) pathways [87,104].

**Figure 2:**
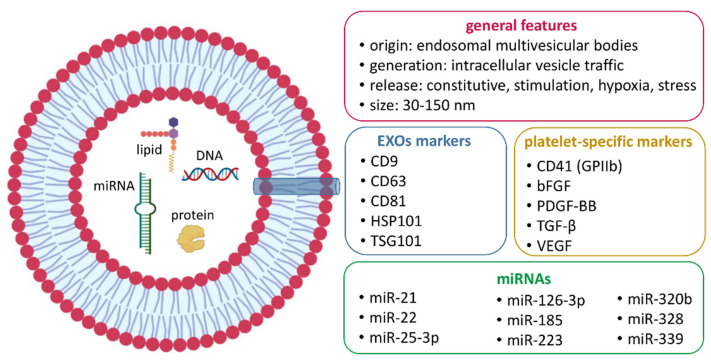
Main characteristics of P-EXOs as detected by transmission electron microcopy (TEM), flow cytometry (FC), Western blot (WB), dynamic light scattering (DLS), and nanoparticle-tracking analysis (NTA).

Figure 2 schematically illustrates the main characteristics of P-EXOs detected through the described methodologies.

## 5. Role of P-EXOs in AS and Atherothrombosis

The role of platelets in the pathophysiology of atherosclerosis has already been elucidated [105,106]; on the contrary, the role played by P-EXOs has not yet been fully clarified. The few studies conducted so far are shown in Table 1.

P-EXOs were originally thought to promote the immune reaction due to the presence of P-selectin on their membrane surface, which is responsible for EC activation and the consequent recruitment of monocytes [16]. However, while EVs derived from platelets have been demonstrated to activate ECs and monocytes [21,107], a direct atherosclerotic role of P-EXOs has not yet been demonstrated. Several factors complicate the full understanding of the regulatory mechanisms employed by P-EXOs. The isolation and characterization of P-EXOs represent the most critical steps in the P-EXO research field, particularly for downstream analyses and comparability between studies. Indeed, platelets can be activated with different agonists, and P-EXOs can be obtained using several protocols. All these factors cause differences in EXO cargo and thus generate experimental errors. In addition, the isolation protocol and characterization technique of P-EXOs often produce an overlap of both subtypes of EVs released by platelets, P-EXOs and P-MPs, leading to the impossibility of distinguishing between them.

Recently, Srikanthan and colleagues demonstrated that P-EXOs affect two essential mechanisms of AS and atherothrombosis by acting on macrophages and platelets [108]. In both cell types, P-EXOs reduce CD36 expression through enhanced ubiquitination and proteasome degradation. In macrophages, the decreased CD36 expression is correlated with a reduced uptake of the harmful cholesterol ox-LDL and, consequently, with the inhibition of foam cell formation. In platelets, CD36 mediates their adhesion to the exposed collagen in damaged vessels. P-EXOs are able to reduce platelet aggregation and adhesion to collagen and mitigate platelet reactivity in an FeCl_3_-induced carotid artery thrombosis model in mice.

In addition to directly inhibiting platelet activation and adhesion, P-EXOs could transfer into VSMCs where they reduce the expression of platelet-derived growth factor receptor-beta (PDGFRb) [109]. The result of decreased PDGFRb expression is the inhibition of VSMC proliferation and the promotion of their apoptosis. An miRNA analysis revealed that thrombin-activated P-EXOs carry high levels of miR-223, miR-339, and miR-21, which are associated with platelet activation, and that PDGFRb is a target gene of the three miRNAs [110,111].

**Table 1:** Role of P-EXOs in AS and atherothrombosis.

Platelet Source	P-EXOs Isolation Method	Study Model	Effect	Ref.
Platelets activated with 0.01 U thrombin O/N in PBS ^a^ or 10 µM calcium ionophore for 1 h in PBS	DC ^b^ (17,000× *g* for 90 min, then 110,000× *g* for 2 h)	in vitro cell culture in vitro thrombogenesis carotid artery thrombosis in mice	reduced CD36 expression by macrophages, that in turn inhibits the entry of ox-LDL ^c^ and cholesterol into these cells, limiting their transformation into foam cells reduced platelet aggregation and adhesion to collagen, that in turn suppresses platelet thrombosis	[108]
Platelets activated with 1 U/mL thrombin for 1 h at 37 °C	DC (5000× *g* for 20 min at RT ^d^, 20,000× *g* for 40 min at 4 °C, then 120,000× *g* for 70 min at 4 °C)	in vitro cell culture carotid artery tandem stenosis in ApoE^−/−^ mice	reduced expression of PDGFRb ^e^ in VSMCs ^f^, leading to inhibition of their proliferation PDGFRb is a target gene of miR-223, miR-339 and miR-21, which are enriched in thrombin-stimulated P-EXOs	[109]
Platelets activated with 1 U/mL thrombin for 30 min at 37 °C	DC (200× *g* for 12 min at RT, 900× *g* for 10 min, 20,000× *g* for 30 min, then 120,000× *g* for 70 min)	in vitro cell culture carotid artery tandem stenosis in ApoE^−/−^ mice	reduced expression of ICAM-1 ^g^ in ECs ^h^ via regulation of the NF-kB ^i^ and MAPK ^l^ pathways mediated by miR-223	[104]
Platelets activated with 0.1 U/mL thrombin for 30 min at 37 °C	commercial kit (ExoQuick™ Exosome Precipitation Solution, System Biosciences) O/N at 4 °C, then 1500× *g* for 30 min	in vitro cell culture ApoE^−/−^ mouse model of AS ^m^	reduced expression of ADAM10 ^n^ in ECs via regulation of the NF-kB pathway mediated by miR-25-3p	[87]
Plasma of patients with CeVD ^o^ and matched control subjects	commercial kit (ExoQuick™ Exosome Precipitation Solution, System Biosciences) O/N at 4 °C, then 1500× *g* for 30 min at 4 °C	ELISA immunoassays	P-EXOs of patients with CeVD contain elevated levels of platelet proteins relevant to AS, such as PDGF-AA ^p^, GPVI ^r^, ILK-1 ^s^, HMGB1 ^t^, CXCL-4, and TSP-1 ^v^, when compared to control subjects	[112]
Platelets activated with 30 nM human plasma-derived thrombin or 0.3 µM human collagen for 30 min at 37 °C	commercial kit (ExoQuick™ Exosome Precipitation Solution, System Biosciences) O/N at 4 °C, then 1500× *g* for 30 min at 4 °C	ELISA immunoassays	aspirin inhibits thrombin- and collagen-induced increases in P-EXO cargo levels of CXCL-4, CXCL-7, and HMGB1, without altering the total levels of plasma P-EXOs	[113]

^a^ phosphate-buffered saline; ^b^ differential centrifugation; ^c^ oxidized lipoprotein; ^d^ room temperature; ^e^ platelet-derived growth factor receptor-beta; ^f^ vascular smooth muscle cells; ^g^ intercellular adhesion molecule-1; ^h^ endothelial cells; ^i^ nuclear factor kappa B; ^l^ mitogen-activated protein kinase; ^m^ atherosclerosis; ^n^ A disintegrin and metalloproteinase domain 10; ^o^ cerebrovascular disease; ^p^ platelet-derived growth factor; ^r^ glycoprotein VI; ^s^ integrin-linked kinase-1; ^t^ high mobility group box-1; ^v^ thrombospondin-1.

As highlighted throughout the text, endothelial dysfunction represents an essential contributor to the pathogenesis of AS. The research group of Li and co-workers investigated the role of P-EXOs in ECs during inflammation-induced thrombosis [104]. The authors found that thrombin-activated P-EXOs release high amounts of miR-223, which in turn inhibits ICAM-1 expression in ECs, and they demonstrated that miR-223 might prevent EC inflammation by regulating the NF-kB and MAPK pathways. To further explore the involvement of P-EXOs in endothelial injury, Yao and colleagues found that thrombin-activated P-EXOs transport high levels of miR-25-3p, which is responsible for reducing EC inflammation induced by ox-LDL as well as lipid deposition, thus inhibiting AS progression. In particular, the authors demonstrated that miR-25-3p targets the A disintegrin and metalloproteinase domain 10 (ADAM10) gene, inhibiting its expression, via the NF-kB pathway, and down-regulates the pro-inflammatory mediators IL-1β, IL-6, TNF-α, as well as triglycerides and total cholesterol [87].

Apart from miRNAs, the potential value of P-EXO cargo proteins as biomarkers of AS has also been investigated. In their study, Goetzl and co-workers measured elevated plasma levels of platelet biomarkers relevant to AS in the P-EXOs of patients with cerebrovascular disease (CeVD) compared to those of matched control subjects [112]. Specifically, PDGF-AA, glycoprotein VI (GPVI), integrin-linked kinase-1 (ILK-1), high mobility group box-1 (HMGB1), chemokine CXCL-4, and thrombospondin-1 (TSP-1) were found to be significantly higher in the P-EXOs of these patients than in healthy subjects. The same research group contextually explored the effects of aspirin, an antiplatelet drug commonly used for preventing CVDs, on P-EXO secretion and cargo levels [113]. Aspirin consumption significantly reduced thrombin- and collagen-induced increases in the P-EXO cargo levels of the chemokines CXCL-4 and CXCL-7 and HMGB1, but did not alter the GPVI level or the total amount of released P-EXOs. HMGB1 is a critical mediator of thrombosis: after being released from first-responding platelets, the protein achieves concentrations that activate and aggregate other platelets, thus initiating a cascade of platelet thrombogenesis [114]. The reduction of the HMGB1 level observed in P-EXOs indicates that aspirin therapy, despite an increased risk of bleeding, might limit platelet contributions to AS and atherothrombosis.

## 6. Conclusions

The pathogenesis of AS progresses through the communication of different cell types. Platelets are considered major players from the onset and progression of AS to thrombus formation after plaque rupture. Platelets exert these important functions by interacting with ECs, leukocytes, VSMCs, as well as with native and modified LDL. Most of these interactions are thought to be mediated by the EVs released from platelets. While the role of P-MPs in AS has been extensively investigated over the past few years, very little attention has been paid to P-EXOs in the same process.

The results of the studies published so far provide evidence that P-EXOs have a role in AS. In particular, thrombin-activated P-EXOs seem to inhibit macrophage foam cell formation, reduce platelet activation, adhesion to collagen, and aggregation, block VSMC proliferation, and protect ECs through multiple anti-inflammatory miRNAs. In addition to miRNAs, elevated plasma levels of proteins implicated in AS have been measured in the P-EXOs of patients with CeVD.

Overall, these data provide new insights into the biochemical nature of P-EXOs, unveil their contribution to AS pathogenesis, and provide a basis for the future use of these vesicles and their transported cargo as biomarkers for the diagnosis and prognosis of AS. However, these findings derive from the analysis of too few studies; consequently, they are not absolute and need to be confirmed by further investigations. The standardization of methodologies and technologies to study P-EXOs will undoubtedly be a mandatory prerequisite for an accurate comprehension of P-EXO functions, for validating their associated biomarkers and, hopefully, for considering possible therapeutic strategies.
